# Maternal glycemic status during pregnancy and mid-childhood plasma amino acid profiles: findings from a multi-ethnic Asian birth cohort

**DOI:** 10.1186/s12916-023-03188-9

**Published:** 2023-11-29

**Authors:** Mengjiao Liu, Shiao-Yng Chan, Johan G. Eriksson, Yap Seng Chong, Yung Seng Lee, Fabian Yap, Mary Foong-Fong Chong, Mya Thway Tint, Jiaxi Yang, David Burgner, Cuilin Zhang, Ling-Jun Li

**Affiliations:** 1 School of Public Health, Nanchang University, Jiangxi, China; 2 Jiangxi Provincial Key Laboratory of Preventive Medicine, Nanchang University, Jiangxi, China; 3 Department of Obstetrics & Gynaecology, Yong Loo Lin School of Medicine, National University of Singapore, Singapore, Singapore; 4 Human Potential Translational Research Programme, Yong Loo Lin School of Medicine, National University of Singapore, Singapore, Singapore; 5 Department of General Practice and Primary Health Care, University of Helsinki, Helsinki, Finland; 6 Folkhälsan Research Center, Helsinki, Finland; 7 Departments of Pediatrics, Yong Loo Lin School of Medicine, National University of Singapore, Singapore, Singapore; 8 Division of Pediatric Endocrinology, Khoo Teck Puat-National University Children’s Medical Institute, National University Hospital, National University Health System, Singapore, Singapore; 9 Departments of Pediatrics, and Diagnostic and Interventional Imaging, KK Women’s and Children’s Hospital, Singapore, Singapore; 10 Graduate Medical School, Duke-National University of Singapore, Singapore, Singapore; 11 Lee Kong Chian School of Medicine, Nanyang Technological University, Singapore, Singapore; 12 Saw Swee Hock School of Public Health, National University of Singapore, Singapore, Singapore; 13 Agency for Science, Technology and Research (A*STAR), Singapore Institute for Clinical Sciences (SICS), Singapore, Singapore; 14 Global Centre for Asian Women’s Health, Yong Loo Lin School of Medicine, National University of Singapore, Singapore, Singapore; 15 Asia Centre for Reproductive Longevity & Equality, Yong Loo Lin School of Medicine, National University of Singapore, Singapore, Singapore; 16 Murdoch Children’s Research Institute, Melbourne, VIC Australia; 17 Department of Paediatrics, The University of Melbourne, Melbourne, VIC Australia; 18 Department of Paediatrics, Monash University, Melbourne, Australia; 19 Department of Nutrition, Harvard T.H. Chan School of Public Health, Boston, MA USA

**Keywords:** Maternal glycemia, Gestational diabetes mellitus, Amino acids, Branched-chain amino acids, Population-based cohort

## Abstract

**Background:**

Increasing maternal glycaemia across the continuum during pregnancy may predispose offspring to subsequent cardiometabolic risk later in life. However, evidence of long-term impacts of maternal glycemic status on offspring amino acid (AA) profiles is scarce. We aimed to investigate the association between maternal antenatal glycaemia and offspring mid-childhood amino acid (AA) profiles, which are emerging cardiometabolic biomarkers.

**Methods:**

Data were drawn from the Growing Up in Singapore Towards healthy Outcomes (GUSTO) study, a multi-ethnic Asian birth cohort. A subset of 422 mother–child dyads from the GUSTO study, who was followed from early pregnancy to mid-childhood, was included. Mothers underwent an oral glucose tolerance test (OGTT) at 26–28 weeks gestation, with fasting and 2-h plasma glucose concentrations measured and gestational diabetes mellitus (GDM) diagnosed per WHO 1999 guidelines. Offspring fasting plasma samples were collected at mean age 6.1 years, from which AA profiles of nine AAs, alanine, glutamine, glycine, histidine, isoleucine, leucine, valine, phenylalanine, and tyrosine were measured. Total branched-chain amino acids (BCAAs) were calculated as the sum of isoleucine, leucine, and valine concentrations. Multi-variable linear regression was used to estimate the association of maternal glycemic status and offspring mid-childhood AA profiles adjusting for maternal age, ethnicity, maternal education, parity, family history of diabetes, ppBMI, child sex, age and BMI *z*-scores.

**Results:**

Approximately 20% of mothers were diagnosed with GDM. Increasing maternal fasting glucose was significantly associated with higher offspring plasma valine and total BCAAs, whereas higher 2-h glucose was significantly associated with higher histidine, isoleucine, valine, and total BCAAs. Offspring born to mothers with GDM had higher valine (standardized mean difference 0.27 SD; 95% CI: 0.01, 0.52), leucine (0.28 SD; 0.02, 0.53), and total BCAAs (0.26 SD; 0.01, 0.52) than their counterparts. Inconsistent associations were found between maternal GDM and other amino acids among offspring during mid-childhood.

**Conclusions:**

Increasing maternal fasting and post-OGTT glucose concentrations at 26–28 weeks gestation were significantly associated with mid-childhood individual and total BCAAs concentrations. The findings suggest that elevated maternal glycaemia throughout pregnancy, especially GDM, may have persistent programming effects on offspring AA metabolism which were strongly associated with adverse cardiometabolic profiles at mid-childhood.

**Supplementary Information:**

The online version contains supplementary material available at 10.1186/s12916-023-03188-9.

## Background

Maternal glycemic status during pregnancy is closely related to short- and long-term offspring outcomes [1]. Gestational diabetes mellitus (GDM), a state of glucose intolerance occurring during pregnancy without pre-existing diabetes, is a well-established early life risk factor for adverse child cardiometabolic health outcomes, such as the increased risk of metabolic disorders (e.g., obesity, metabolic syndrome, abnormal glucose metabolism) and hypertension [1]. However, the mechanistic and biological pathways underlying these risks remain unclear. It has been postulated that women who developed GDM might have already displayed a series of subclinical metabolic variations as early as in preconception and early pregnancy [2], which may lead to subsequent cardiometabolic in-utero programming in offspring, where the relevant phenotypes may only clinically manifest later in life.

Emerging evidence suggests that maternal metabolomic profiles, including the lipidome and amino acid (AA) concentrations [3], are altered in GDM, which would be expected to have knock-on effects on the intrauterine environment. For example, a meta-analysis of eight studies including 432 participants reported that mothers with GDM had higher concentrations of individual and total branched-chain amino acids (BCAAs), namely leucine, isoleucine, and valine, compared with those without GDM [4]. Such changes were also seen in offspring born to mothers with GDM. Maternal GDM is also associated with infant cord blood AAs (e.g., elevation in valine, isoleucine, leucine, phenylalanine, glutamate, proline, and alanine) [5, 6]. AAs are associated with cardiometabolic health; for example, alanine, glutamine, and glycine were found to be protective [7], while phenylalanine, tyrosine, and BCAAs were associated with adverse cardiometabolic health [8]. Specific AAs have been associated with cardiovascular outcomes, suggesting their pivotal roles in the pathogenesis of cardiovascular diseases (CVD). For instance, plasma glycine has demonstrated an inverse association with the risk of acute myocardial infarction (hazard ratio [HR] per standard deviation [SD]: 0.89; 95% CI, 0.82–0.98), after adjusting for major traditional CVD risk factors [9]. In addition, a systematic review and meta-analysis included 10 prospective studies involving 43,895 participants and reported a 10% higher risk of CVD per study-specific SD for isoleucine (pooled relative risk 1.10, 95% CI 1.03–1.18) [10]. Therefore, maternal glycemic status during pregnancy may influence offspring cardiometabolic programming via AA metabolism. However, studies to date have been mainly limited to cord blood AAs. Evidence regarding the long-term influence of GDM on offspring AAs is sparse, and in particular, data on maternal glycemia across the continuum (in the absence of GDM) may also contribute to offspring AA metabolism.

To address these knowledge gaps, we aimed to investigate the influence of maternal glycemia during mid-late pregnancy on offspring AAs in mid-childhood and associations of AA profiles and concurrent cardiometabolic risk in an ongoing multi-ethnic birth cohort in Singapore. We hypothesized that offspring born to mothers with abnormal glycemic status during pregnancy were more likely to have higher concentrations of AAs related to cardiometabolic risk (such as BCAAs) in mid-childhood.

## Methods

### Study population

The Growing Up in Singapore Towards healthy Outcomes (GUSTO) study recruited pregnant women aged ≥ 18 years from Singapore’s two major public maternity hospitals (National University Hospital and KK Women’s and Children’s Hospital) between June 2009 and September 2010. Inclusion criteria for pregnant women were as follows: (1) Singaporean residents aged 18 years and above, (2) attending either KK Women’s and Children’s Hospital (KKH) or National University Hospital (NUH), and (3) intending to deliver and reside in Singapore for the next 5 years. Of 3751 women screened, 2034 met eligibility criteria, and 1344 were recruited (response rate 66.1%). These women gave birth to 1098 singleton infants. GUSTO mothers and children have been followed up since birth. At postpartum year-6 follow-up, 953 (86.8%) offspring were assessed, of whom 460 (48.3%) provided blood samples. Participants were included in the analytic sample if they had maternal glucose data and at least one AA as an outcome (*n* = 422, see Additional file 2: Fig. S1). Informed written consent was obtained from the women at the study entry, and the National Healthcare Group Domain Specific Review Board and SingHealth Centralized Institutional Review Board approved the study. Detailed study designs and recruitment have been published elsewhere [11].

### Maternal glycemic status assessment

At 26–28 weeks of gestation, pregnant women without pre-existing diabetes underwent a 2-h 75 g oral glucose tolerance test (OGTT) after an overnight fast [11]. Fasting and 2-h post-challenge venous blood samples were collected in fluoride-containing tubes, and glucose concentrations were assessed quantitatively (Advia 2400 Chemistry system and Beckman LX20 Pro analyzer). Women were diagnosed with GDM based on World Health Organization’s (WHO) 1999 guidelines (fasting plasma glucose ≥ 7.0 mmol/L and/or 2-h glucose ≥ 7.8 mmol/L]) [12]. Mothers who were diagnosed as having GDM were either managed by diet and/or medication (i.e., metformin and insulin) according to standard protocols practiced at study sites.

### Mid-childhood offspring amino acids and other biomarkers assessments

We collected fasted peripheral blood from 460 children at approximately six years of age. Blood samples were immediately fractionated, aliquoted, and stored at – 80 °C until transported on dry ice to Nightingale Health (Helsinki, Finland) for further analyses [13]. Circulating metabolite concentrations were quantified using an automated nuclear magnetic resonance (NMR)-based high throughput metabolomics platform [14]. The software of NMR platform undertakes automatic quality control [14]. After quality control, metabolomic data were available for 457 children. As the AAs we investigated were part of the metabolomic profile measured by the Nightingale platform, our analysis is confined to the nine AAs analyzed in this study, which included alanine, glutamine, glycine, histidine, isoleucine, leucine, valine, phenylalanine, and tyrosine. We calculated total BCAAs as the sum of isoleucine, leucine, and valine concentrations and aromatic AAs as the sum of phenylalanine and tyrosine [14]. Also, among the metabolomic data, fatty acids were measured. Measurements of other cardiometabolic measures implicated in the development of cardiometabolic diseases, such as homeostasis model assessment of insulin resistance (HOMA-IR), interleukin 6 (IL-6), high-sensitivity C-reactive protein (hsCRP), low-density lipoprotein (LDL) cholesterol, high-density lipoprotein (HDL) cholesterol, and triglyceride, are shown in Additional file 1.

### Covariates

At recruitment, maternal age, ethnicity, highest education level, parity, pre-pregnancy weight, and family history of diabetes were obtained through interviewer-administered questionnaires. Maternal height was measured using SECA 213 Stadiometer (SECA Corp, Hamburg, Germany) [15]. Maternal weight during pregnancy was measured using SECA 803 Weighing Scale (SECA Corp, Hamburg, Germany). Body weight and height were used to calculate pre-pregnancy body mass index (ppBMI, in kg/m^2^). Gestational weight gain (GWG) was calculated as the difference between the final measured weight before delivery and self-reported pre-pregnancy weight. Excessive GWG was classified according to the Institute of Medicine recommendation [16]. Information on dietary intakes of women was collected at 26–28 weeks of gestation using 24-h recalls and 3-day food diaries, from which diet quality (score range: 0–100) was measured by the Healthy Eating Index [17].

Date of child age, weight, and height were collected at their year-6 follow-up. Child BMI was calculated, and sex-specific BMI *z*-scores were generated using the WHO references [18]. Since some AAs, such as BCAAs, cannot be synthesized from other metabolites by the human body but are derived from diet intake [19], we also considered the influence of food intake on blood AA concentrations. At the year-5 visit, about 1 year before AA measurements, child protein intake in the previous month was assessed using an interviewer-administered 112 food items semi-quantitative food frequency questionnaire completed by the caregivers [20].

### Statistical analyses

Distributions for all variables were checked for skewness and kurtosis. Maternal fasting glucose, 2-h glucose concentrations, and all offspring mid-childhood AAs were analyzed as continuous variables, and GDM status was analyzed as a binary variable (present/absent). Comparisons of characteristics between GDM and non-GDM participants were analyzed by Student’s *t*-test, non-parametric comparison test, or *χ*^2^-test when applicable.

Multi-variable linear regressions were applied to examine the associations of maternal fasting glucose concentration at test, 2-h glucose concentrations, and GDM diagnosis with child AA profiles, using three models: unadjusted model; model 1, adjusting for maternal age, ethnicity, maternal education, parity, family history of diabetes, ppBMI and child sex; and model 2: model 1 and additionally adjusting for child age and BMI *z*-score at mid-childhood. Furthermore, we tested the interactions between maternal GDM status with maternal age and ppBMI, respectively.

In sensitivity analysis, we considered potential confounders that were associated with maternal glycemic levels and child AAs. Linear regression models were performed with additional adjustments for excessive GWG, hypertension diagnosed during pregnancy, maternal Health Eating Index during pregnancy, child fatty acids, and child protein intake assessed at mid-childhood in addition to model 2. As child birthweight and gestational age at birth are recognized as significant risk factors for child cardiometabolic health, we conducted a sensitivity analysis with further adjustment for these two variables, in addition to model 2. Also, GDM mothers treated with medication or missing treatment data were excluded to investigate the direct effect without medication intervention between GDM and child AAs concentrations. We further corrected for multiple comparison using the Benjamini–Hochberg method to control false-discovery rate (FDR) [21].

To assess the potential relationships among mid-childhood offspring plasma AAs and cardiometabolic risks within our cohort, partial Spearman rank correlation was performed a posteriori after adjusting for maternal age, ppBMI, GDM status, GWG, child sex, age and BMI *z*-score at mid-childhood. The cardiovascular phenotypes (i.e., SBP, DBP, carotid intima-media thickness, pulse wave velocity, and augmentation index) and metabolic phenotypes (i.e., child HOMA-IR, IL-6, hsCRP, total fatty acids, total polyunsaturated fatty acids, total saturated fatty acids, total monounsaturated fatty acids, LDL cholesterol, HDL cholesterol, and triglyceride) were assessed accordingly.

Analyses were performed in Stata 16.0 SE (StataCrop LP, TX, USA). For all analyses, we standardized exposures (fasting glucose and 2-h glucose) and outcomes (each AA) to present effect size in standardized regression coefficients. *P* values and 95% confidence intervals (CIs) are presented accordingly. A significant *P*-value (two-tailed) was defined as < 0.05.

## Results

Characteristics of the 422 mother–child dyads are presented in Table 1. Comparisons between sample characteristics with or without amino acids were presented in Additional file 2: Table. S1. The mean age of mothers was 31.0 ± 5.1 years, 56.4% of whom were Chinese, and 43.6% were Malay or Indian. Mean maternal fasting glucose and 2-h fasting glucose were 4.3 ± 0.5 mmol/L and 6.6 ± 1.5 mmol/L, respectively. According to the 1999 WHO criteria, 19.7% of mothers in the study cohort were diagnosed with GDM, comparable to the GDM incidence (18.9%) reported in the whole GUSTO cohort [22] and 23.8% in Singapore reported by the International Diabetes Federation 2021 [23]. Except for parity, family history of diabetes and hypertensive disorders during pregnancy, most of the maternal characteristics were different between GDM and non-GDM mothers, including maternal age, ethnicity, education, ppBMI, total GWG, glucose concentrations (fasting, 2-h, and GDM), and Healthy Eating Index (Table 1).

**Table 1:** GUSTO analytic sample characteristics (*n* = 422)

Variables	All mothers	GDM	Non-GDM	*P*-value
	**Mean (SD)/%**	**Mean (SD)/%**	**Mean (SD)/%**	
***Mothers***				
Mother age (whole years)	31.0 (5.1)	33.1 (5.0)	30.5 (5.1)	** < 0**.**001**
Ethnicity				
Chinese	56.4	59.2	56.4	** < 0**.**01**
Malay	27.5	13.6	30.3	
Indian	16.1	27.2	13.3	
Highest education				**0**.**03**
Below university	64.7	54.3	67.3	
University	35.3	45.7	32.7	
Parity				0.16
0	38.6	32.1	40.6	
≥ 1	61.4	67.9	59.4	
Pre-pregnancy BMI (kg/m^2^)	22.77 (4.25)	23.65 (4.06)	22.5 (4.22)	**0**.**03**
Categorical ppBMI (WHO)^a^				0.06
Normal and underweight	61.4	53.1	64.2	
Overweight or obese	38.6	46.9	35.8	
Total gestational weight gain (kg)	13.81 (5.19)	11.8 (4.76)	14.28 (5.17)	** < 0**.**001**
Excessive GWG (according to IOM, %)	41.6	38.8	49.8	0.08
Glycemia at 26 weeks’ gestation				
Fasting glucose, mmol/L	4.33 (0.46)	4.58 (.58)	4.27 (.4)	** < 0**.**001**
Two-hour glucose, mmol/L	6.56 (1.48)	8.82 (.97)	6.01 (.97)	** < 0**.**001**
Gestational diabetes (according to 1999 WHO, %)	19.7			
Gestational diabetes treatment (%)				
Diet treated		88.9		
Diet and insulin treated		8.6		
Not treated		2.5		
Family history of diabetes (yes, %)	31.0	38.3	29.4	0.12
Hypertensive disorder (yes, %)	7.8	13.6	6.1	0.08
Health eating index	53.28	58.21 (13.06)	52.07 (13.49)	** < 0**.**001**
***Children***				
Child age at year 6 visit	6.1 (0.1)	6.05 (.1)	6.05 (.09)	0.64
Sex (girls, %)	47.6	49.4	47.6	0.61
Child BMI (kg/m^2^)	15.46 (2.09)	15.36 (1.73)	15.49 (2.16)	0.68
Child BMI *z*-score	− 0.04 (1.28)	− 0.09 (1.14)	− 0.03 (1.31)	0.77
Alanine (μmol/L)	266.6 (61.67)	256.02 (58.22)	268.46 (62.22)	0.11
Glutamine (μmol/L)	645.79 (65.64)	640.32 (68.55)	646.99 (65.57)	0.38
Glycine (μmol/L)	182.46 (42.87)	175.78 (36.38)	183.64 (44.29)	0.12
Histidine (μmol/L)	78.2 (10.78)	79.28 (10.17)	77.83 (10.93)	0.36
Isoleucine (μmol/L)	45.4 (10.81)	46.26 (10.85)	45.18 (10.89)	0.48
Leucine (μmol/L)	90.7 (16.6)	94.69 (18.36)	89.73 (16.11)	**0**.**02**
Valine (μmol/L)	212.32 (30.8)	218.72 (28.88)	210.72 (31.34)	**0**.**04**
Total branched-chain amino acids (μmol/L)	348.42 (54.53)	359.67 (54.16)	345.63 (54.77)	**0.04**
Phenylalanine (μmol/L)	50.34 (8.8)	50.58 (7.36)	50.08 (8.95)	0.68
Tyrosine (μmol/L)	63.6 (11.17)	63.38 (11.15)	63.39 (11.16)	0.70
Total aromatic amino acids (mmol/L)	113.95 (16.66)	113.96 (15.03)	113.47 (16.79)	0.96

^a^BMI category was based on WHO Asian population cutoffs: BMI < 23 kg/m^2^ as normal and underweight; BMI ≥ 23.0kg/m^2^ as overweight and obesity

A significant *P*-value (two-tailed) was defined as < 0.05

The mean age and SD of included offspring at mid-childhood were 6.1 ± 0.1 years; 47.6% were girls. Summary statistics for nine amino acids, total (BCAAs), and total aromatic AAs were shown in Table 1. There were significant differences in child leucine, valine, and total BCAAs concentrations between GDM and non-GDM mothers (*P* < 0.05 in *t*-test).

### Maternal glycemic levels during pregnancy and offspring mid-childhood AAs profile

In the unadjusted models, higher maternal 26–28-week fasting glucose concentration was associated with higher offspring mid-childhood plasma valine, total BCAAs, tyrosine, and aromatic AAs (Table 2). After the full adjustment (model 2), only the association with offspring mid-childhood valine remained significant, while total BCAAs, tyrosine, and aromatic AAs attenuated. Per SD (0.46 mmol/l) increase in maternal fasting glucose at 26–28 weeks of gestation was associated with a 0.12-SD (95% CI: 0.02, 0.22) increment in offspring plasma valine in mid-childhood.

**Table 2:** Liner regression analyses of mothers’ glycemic level with child amino acids at mid-childhood

	**Raw model**		**Adjusted model 1**		**Adjusted model 2**	
	Effect size (95% CI)	*p*	Effect size (95% CI)	*p*	Effect size (95% CI)	*p*
***Fasting glucose***						
Alanine	0.02 (− 0.07, 0.12)	0.64	0.02 (− 0.08, 0.13)	0.63	0.01 (− 0.09, 0.11)	0.87
Glutamine	0.08 (− 0.02, 0.18)	0.10	0.06 (− 0.04, 0.16)	0.22	0.06 (− 0.04, 0.16)	0.23
Glycine	− 0.00 (− 0.10, 0.09)	0.96	− 0.00 (− 0.10, 0.10)	0.99	0.00 (− 0.10, 0.11)	0.93
Histidine	0.05 (− 0.05, 0.14)	0.34	0.05 (− 0.05, 0.16)	0.32	0.06 (− 0.05, 0.16)	0.30
Isoleucine	0.09 (− 0.00, 0.19)	0.06	0.10 (− 0.01, 0.20)	0.07	0.08 (− 0.03, 0.18)	0.14
Leucine	0.08 (− 0.01, 0.18)	0.09	0.08 (− 0.02, 0.18)	0.13	0.06 (− 0.04, 0.16)	0.27
Valine	**0**.**13 (0**.**03, 0**.**23)**	**0**.**01**	**0**.**14 (0**.**04, 0**.**24)**	**0**.**01**	**0**.**12 (0**.**02, 0**.**22)**	**0**.**02**
Total BCAAs	**0**.**12 (0**.**02, 0**.**22)**	**0**.**02**	**0**.**12 (0**.**02, 0**.**22)**	**0**.**02**	0.10 (− 0.00, 0.20)	0.05
Phenylalanine	0.09 (− 0.01, 0.18)	0.07	0.09 (− 0.01, 0.19)	0.08	0.06 (− 0.04, 0.15)	0.26
Tyrosine	**0**.**10 (0**.**01, 0**.**20)**	**0**.**04**	0.07 (− 0.03, 0.18)	0.14	0.05 (− 0.05, 0.15)	0.31
Aromatic AAs	**0**.**12 (0**.**02, 0**.**21)**	**0**.**02**	0.10 (− 0.00, 0.20)	0.05	0.06 (− 0.03, 0.16)	0.19
***Two-hour glucose***						
Alanine	− 0.06 (− 0.16, 0.03)	0.20	− 0.02 (− 0.13, 0.08)	0.65	− 0.02 (− 0.12, 0.08)	0.67
Glutamine	− 0.05 (− 0.15, 0.04)	0.28	− 0.04 (− 0.14, 0.06)	0.41	− 0.04 (− 0.14, 0.06)	0.47
Glycine	− 0.06 (− 0.16, 0.04)	0.23	− 0.06 (− 0.17, 0.04)	0.23	− 0.06 (− 0.16, 0.05)	0.27
Histidine	**0**.**10 (0**.**00, 0**.**20)**	**0**.**04**	**0**.**12 (0**.**01, 0**.**22)**	**0**.**03**	**0**.**13 (0**.**02, 0**.**23)**	**0**.**02**
Isoleucine	0.08 (− 0.01, 0.18)	0.09	0.10 (0.00, 0.21)	0.05	0.10 (0.00, 0.21)	0.05
Leucine	**0**.**15 (0**.**06, 0**.**25)**	**0**.**00**	**0**.**15 (0**.**04, 0**.**25)**	**0**.**01**	**0**.**14 (0**.**04, 0**.**24)**	**0**.**01**
Valine	**0**.**13 (0**.**04, 0**.**23)**	**0**.**01**	**0**.**13 (0**.**03, 0**.**24)**	**0**.**01**	**0**.**13 (0**.**03, 0**.**23)**	**0**.**01**
Total BCAAs	**0**.**14 (0**.**04, 0**.**24)**	**0**.**01**	**0**.**14 (0**.**04, 0**.**24)**	**0**.**01**	**0**.**14 (0**.**04, 0**.**24)**	**0**.**01**
Phenylalanine	0.06 (− 0.03, 0.16)	0.20	0.09 (− 0.02, 0.19)	0.10	0.09 (− 0.01, 0.19)	0.07
Tyrosine	0.04 (− 0.06, 0.14)	0.43	0.04 (− 0.06, 0.14)	0.47	0.05 (− 0.05, 0.14)	0.36
Aromatic AAs	0.06 (− 0.04, 0.16)	0.22	0.07 (− 0.03, 0.17)	0.17	0.08 (− 0.02, 0.18)	0.11

Adjusted model 1 covariates: maternal age, child ethnicity, mothers’ highest education, parity, family history of diabetes, child sex, and pre-pregnancy body mass index

Adjusted model 2 covariates: model 1 further added in child year-6 body mass index *z*-score and age

A significant *P*-value (two-tailed) was defined as < 0.05

Higher maternal 26–28-week 2-h glucose concentration was associated with higher offspring mid-childhood plasma histidine, leucine, valine, and total BCAAs in three models. In the fully adjusted model 2, per SD (1.48 mmol/l) increase in maternal 2-h glucose was associated with a 0.14-SD (0.04, 0.24) increment in offspring total BCAAs and individual BCAAs in mid-childhood (leucine: 0.14-SD, 0.04, 0.24; valine: 0.13-SD, 0.03, 0.23; isoleucine: 0.10-SD, 0.00, 0.21).

**Fig. 1:**
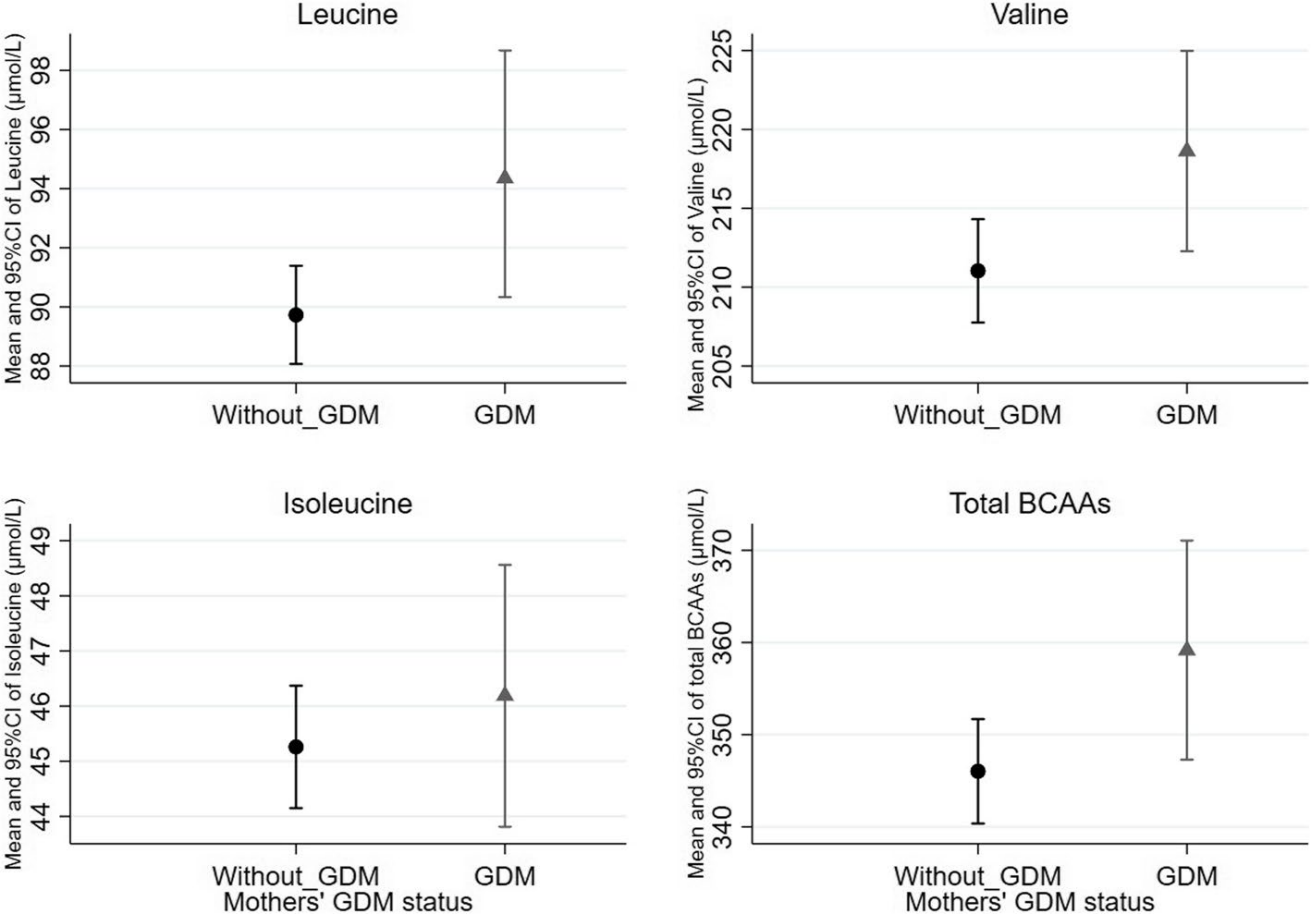
Means and 95%CI of mid-childhood leucine, valine, and total BCAAs by maternal GDM status

Offspring born to mothers with GDM had significantly higher plasma leucine, valine, and total BCAAs than those born to mothers without GDM, which was consistent across the raw model, adjusted model 1 and adjusted model 2 with similar magnitude of effect size (Table 3 and Fig. 1). For example, in the fully adjusted model 2 (also presented in Fig. 1), offspring born to mothers with GDM had increments of 0.28-SD in plasma concentrations of leucine (0.02, 0.53), 0.27-SD in valine (0.01, 0.52), and a 0.26-SD in total BCAAs (0.01, 0.52), compared with those born to mothers without GDM (Fig. 1). No associations were found between maternal GDM and other amino acids among offspring during mid-childhood, such as total aromatic AAs, alanine, glutamine, or glycine.

**Table 3:** Liner regression analyses of GDM (1999 WHO) with child amino acids at mid-childhood

	**Raw model**		**Adjusted model 1**		**Adjusted model 2**	
Amino acids	Effect size (95% CI)	*p*	Effect size (95% CI)	*p*	Effect size (95% CI)	*p*
Alanine	− 0.20 (− 0.45, 0.04)	0.11	− 0.16 (− 0.42, 0.09)	0.21	− 0.14 (− 0.39, 0.11)	0.28
Glutamine	− 0.10 (− 0.35, 0.15)	0.42	− 0.12 (− 0.36, 0.13)	0.35	− 0.10 (− 0.35, 0.15)	0.44
Glycine	− 0.18 (− 0.43, 0.06)	0.14	− 0.20 (− 0.46, 0.05)	0.12	− 0.20 (− 0.46, 0.06)	0.13
Histidine	0.14 (− 0.11, 0.38)	0.28	0.14 (− 0.11, 0.40)	0.27	0.18 (− 0.08, 0.44)	0.18
Isoleucine	0.10 (− 0.15, 0.35)	0.43	0.14 (− 0.12, 0.39)	0.30	0.14 (− 0.12, 0.39)	0.30
Leucine	**0**.**30 (0**.**05, 0**.**54)**	**0**.**02**	**0**.**29 (0**.**03, 0**.**54)**	**0**.**03**	**0**.**28 (0**.**02, 0**.**53)**	**0**.**03**
Valine	**0**.**26 (0**.**01, 0**.**51)**	**0**.**04**	**0**.**27 (0**.**01, 0**.**52)**	**0**.**04**	**0**.**27 (0**.**01, 0**.**52)**	**0**.**04**
Total BCAAs	**0**.**26 (0**.**01, 0**.**50)**	**0**.**04**	**0**.**26 (0**.**01, 0**.**52)**	**0**.**04**	**0**.**26 (0**.**01, 0**.**52)**	**0**.**04**
Phenylalanine	0.06 (− 0.18, 0.30)	0.64	0.09 (− 0.17, 0.34)	0.50	0.11 (− 0.13, 0.36)	0.37
Tyrosine	− 0.00 (− 0.25, 0.24)	0.99	− 0.06 (− 0.31, 0.20)	0.67	− 0.03 (− 0.27, 0.21)	0.82
Aromatic AA	0.03 (− 0.21, 0.27)	0.81	0.01 (− 0.24, 0.26)	0.94	0.04 (− 0.20, 0.28)	0.74

Adjusted model 1 covariates: maternal age, child ethnicity, mothers’ highest education, parity, family history of diabetes, child sex, and pre-pregnancy body mass index

Adjusted model 2 covariates: Model 1 further added in child year-6 body mass index *z*-score and age

A significant *P*-value (two-tailed) was defined as < 0.05

No significant interactions were found between maternal GDM status and maternal age or ppBMI in relation to the offspring mid-childhood AAs concentrations (Additional file 2: Table. S2). Sensitivity analysis for all reported associations remained significant after (1) additionally adjusting for excessive GWG, hypertension diagnosed during pregnancy, maternal Healthy Eating Index during pregnancy, and child protein intake at 5-year-old each (Additional file 2: Table. S3-S5); (2) in the sensitivity analysis, where we included further adjustments for child birthweight and gestational age at birth, we observed consistently stronger associations across all models (Additional file 2: Table. S6); and (3) excluding GDM mothers who were treated with medication (*n* = 7) or missing treatment data (*n* = 2, Additional file 2: Table. S7). After multiple testing using FDR correction, the associations of maternal 2-h glucose concentrations and AAs remained statistically significant whereas the significance of the rest associations attenuated (Additional file 2: Table. S8).

### Correlations of mid-childhood AA profiles and cardiometabolic risks

Offspring mid-childhood cardiometabolic characteristics were presented in Additional file 2: Table. S9; only LDL was different between offspring born to GDM or non-GDM mothers. We analyzed the cross-sectional association of each AA, total BCAAs, and aromatic AAs with child cardiometabolic characteristics. AAs like isoleucine, leucine, valine, and total BCAAs were positively associated with mid-childhood cardiometabolic risks like HOMA-IR and hsCRP; whereas AAs like glycine, phenylalanine, and aromatic AAs were inversely associated with cardiometabolic risks like lipid concentrations (Additional file 2: Fig. S2).

## Discussion

In this study of Singaporean mother–child dyads, we reported that increasing maternal glucose concentrations and GDM during mid-late pregnancy was significantly and positively associated with adverse offspring AA profiles at mid-childhood, even after adjusting for child BMI *z*-scores. Higher maternal fasting glucose was associated with higher concentrations of offspring valine and total BCAAs in mid-childhood; whereas higher maternal 2-h glucose was associated with higher concentrations of offspring histidine, isoleucine, valine, and total BCAAs in mid-childhood. Offspring born to mothers with GDM had higher concentrations of leucine, valine, and total BCAAs during mid-childhood compared to mothers with normal glucose concentrations in pregnancy. In addition, isoleucine, leucine, valine, and total BCAAs were positively correlated with concurrent cardiometabolic profile, including HOMA-IR, and hsCRP.

Emerging evidence has underpinned the pivotal role of AAs in the development of CVDs [10, 24, 25]. For instance, a wealth of epidemiological research consistently demonstrated a positive correlation between elevated circulating BCAA levels and cardiometabolic risks (e.g., overweight/obesity, metabolic syndrome, type 2 diabetes) and even CVDs [24, 25]. Findings from the Women’s Health Study, which encompassed 25,994 US women without pre-existing CVD at the outset and entailed a median follow-up period of 18.6 years, reported a direct association between total BCAAs and the incidence of CVD (HR: 1.13; 95% CI: 1.08, 1.18) [26]. Remarkably, this association was comparable with the other association observed between LDL-cholesterol and CVD (per SD. LDL-cholesterol HR, 1.12; 95% CI: 1.07, 1.17) [26]. Both BCAAs and LDL appeared to mediate the path to CVDs by approximately 13% each [27]. These results collectively emphasize the pivotal role of AAs in the development of CVDs.

In addition, a couple of recent systematic review [28] and meta-analysis [4] suggested a strong link between GDM and maternal AA metabolism during pregnancy, particularly BCAAs. Consequently, maternal metabolite changes in response to GDM may further impact postnatal metabolomic programming by transplacental transfer and/or by altering the expression and function of genes involved in AA metabolism in the offspring [29]. For instance, Dani et al. reported that term infants with GDM mothers had higher concentrations of cord serum AAs, such as pyruvate, histidine, alanine, valine, methionine, arginine, and lysine [30]. Infants born to mothers with GDM have elevated meconium metabolites, including AAs (i.e., taurine, phenylalanine, and tyrosine), compared with those born to mothers without GDM [31]. These findings supported that hyperglycemia-induced high concentrations of maternal metabolites could lead to high concentrations of offspring AA metabolism, and based on our findings, the altered offspring AAs may persist into mid-childhood.

Animal studies suggest GDM may change offspring metabolic programming through epigenetic mechanisms. Using a GDM mouse model, Zhu et al. [32] explored the impact of maternal hyperglycemia on fetal pancreatic metabolome in mice of embryonic day 18.5. The metabolome profiling of the fetal pancreas showed altered metabolites in several important pathways, including the BCAAs pathway. Human data have demonstrated an association between maternal GDM exposure and DNA methylation changes in cord blood, placenta, and offspring peripheral blood DNA [33–35]. For example, *PTPRN2* (receptor-type tyrosine-protein phosphatase N2) gene was methylated in cord blood and in whole blood of GDM-exposed offspring at age 10.5 years [35]. These findings suggest epigenetic modifications may be also involved in the long-term programming of AA metabolism and processing in association with GDM.

Elevated circulating concentrations of BCAAs are known risk factors for obesity, type 2 diabetes, insulin resistance, and adverse CVD outcomes, such as stroke, in the general adult population [36]. Emerging evidence also suggests a similar relationship in children with findings that higher BCAA concentrations (including valine, leucine, isoleucine, and downstream intermediates of BCAAs catabolism) were more prevalent in children with obesity compared to those without, and higher circulating BCAAs were also associated with insulin resistance in children with obesity [37]. Our a posteriori analyses observed correlations between BCAAs and mid-childhood HOMA-IR and CRP, which are in keeping with these published findings.

We found that higher maternal fasting glucose and 2-h glucose were associated with higher concentrations of BCAAs and its components, which in turn were associated cross-sectionally with adverse cardiometabolic profiles. These findings suggest a potential opportunity for intervention for future cardiometabolic health risks, given that BCAAs are essential AAs that can only be obtained from the diet. Furthermore, our findings provide evidence of transgenerational metabolic programming due to adverse maternal hyperglycemia, underlined by BCAAs. Future studies are warranted to verify our findings in larger populations with greater race/ethnicity diversity.

The strengths of our study include a relatively large sample size of mother–child pairs with ~ 20% GDM incidence. Since current evidence of maternal hyperglycemia and offspring AAs profile was limited to infancy, our is the first to report a long-term effect of maternal glycemia during pregnancy on offspring AAs at mid-childhood. We used standardized protocols to collect maternal characteristics and a wide range of potential confounders, diagnose GDM, and assess child AAs profile and other biomarkers. We acknowledge some limitations. First, approximately two thirds of offspring were followed up at mid-childhood, of whom 50% had blood samples collected. Therefore, selection bias cannot be excluded. However, there were small differences between participated mother–child dyads with and without complete data (Additional file 2: sTable 1); for example, those without completed data had younger mothers and higher rate of nulliparous and lower Health Eating Index, and there were no differences in child sample characteristics. Second, we recognize that other AAs, which were not covered by this panel, may also play a role in the development of cardiometabolic diseases. These unexamined AAs warrant further investigation. Furthermore, it should be noted that the significance of certain associations pertaining to maternal GDM or fasting glucose concentrations and AAs attenuated following multiple testing. The interpretation of our findings is contingent upon individual researchers' acceptance of the implications of multiple testing. Thirdly, other unmeasured residual confounders such as paternal BMI and environmental factors could have contributed to the associations observed. Fourthly, offspring AAs and cardiometabolic risk factors were measured cross-sectionally, which limited us to directly examine the potential mediating role of AAs in maternal hyperglycemia and child cardiometabolic health. Last but not least, our cohort comprises Asian mother–offspring pairs from a relatively higher socioeconomic status; so, our findings may not be generalizable to more disadvantaged populations or other race/ethnic groups.

## Conclusions

In summary, we observed that increasing maternal glycemia and GDM during 26-28 weeks of gestation were significantly associated with higher concentrations of offspring BCAAs in mid-childhood, which have been implicated in the development of cardiometabolic disorders later in life. Our findings suggest higher maternal glycemia during mid-to-late pregnancy may have initiated the alteration of metabolic programming of the offspring as early as in the womb.

### Supplementary Information

**Additional file 1: sFile 1. **Additional cardiometabolic measures.

**Additional file 2: sFigure 1. **Participant flowchart.** sFigure 2. **Partial spearman correlation analyses of child amino acids and cardiometabolic measures assessed at mid-childhood. **sTable 1.** Compare participant characteristics with or without amino acids. **sTable 2.** GUSTO analytic sample cardiometabolic profiles (*n* = 422). **sTable 3.** Interaction exploration in liner regression analyses of GDM with child amino acids at mid-childhood. **sTable 4.** Sensitivity linear regression analyses of fasting glucose with child amino acids at mid-childhood. **sTable 5.** Sensitivity linear regression analyses of 2-h glucose with child amino acids at mid-childhood. **sTable 6.** Sensitivity linear regression analyses of GDM with child amino acids at mid-childhood. **sTable 7.** Sensitivity linear regression analyses of maternal glucose level with child amino acids at mid-childhood with additional adjustment of child birthweight and gestation age. **sTable 8.** Sensitivity analyses of maternal glucose level with child amino acids at mid-childhood in GDM mothers without medical treatment (*n* = 413). **sTable 9.** Associations of maternal glucose level with child amino acids at mid-childhood, model 2 with FDR corrections.

## Data Availability

Data described in the manuscript, code book, and analytic code will be available upon request pending application and approval of a data-sharing agreement.

## Abbreviations

AA: Amino acid
BCAAs: Branched-chain amino acids
CVD: Cardiovascular disease
CIs: Confidence intervals
GDM: Gestational diabetes mellitus
GUSTO: Growing Up in Singapore Towards healthy Outcomes
GWG: Gestational weight gain
HR: Hazard ratio
HDL: High-density lipoprotein
HOMA-IR: Homeostasis model assessment of insulin resistance
hsCRP: High-sensitivity C-reactive protein
IL-6: Interleukin 6
LDL: Low-density lipoprotein
NMR: Nuclear magnetic resonance
OGTT: Oral glucose tolerance test
ppBMI: Pre-pregnancy body mass index
SD: Standard deviation
SMD: Standardized mean difference
WHO: World Health Organization
