# Supplementary material for: Maternal glycemic status during pregnancy and mid-childhood plasma amino acid profiles: findings from a multi-ethnic Asian birth cohort
Source: BMC Med. 2023 Nov 29;21:472. doi: 10.1186/s12916-023-03188-9 (PMC10688057; doi:10.1186/s12916-023-03188-9)
Supplement: Supplementary file 1 — Additional file 1: sFile 1. Additional cardiometabolic measures. [file 12916_2023_3188_MOESM1_ESM.docx]

**Additional file 1**

**Maternal Glycaemic Status during Pregnancy and Mid-Childhood Plasma Amino Acid Profiles: Findings from A Multi-Ethnic Asian Birth Cohort**

Mengjiao Liu, PhD;^1,2^ Shiao-Yng Chan, PhD;^3,4^ Johan G. Eriksson, MD;^3,4,5,6^ Yap Seng Chong, MD;^3^ Yung Seng Lee, PhD;^7,8^ Fabian Yap, MBBS;^9,10,11^ Mary Foong-Fong Chong, PhD;^12^ Mya Thway Tint, PhD;^4,13^ Jiaxi Yang, PhD^3,14,15^ David Burgner, PhD;^16,17,18^ Cuilin Zhang, PhD;^3,4,14,15,19^ Ling-Jun Li, PhD^3,4,14,15*^

**Affiliations:**

^1^ School of Public Health, Nanchang University, Jiangxi, China

^2^ Jiangxi Provincial Key Laboratory of Preventive Medicine, Nanchang University, Jiangxi, China

^3^ Department of Obstetrics & Gynaecology, Yong Loo Lin School of Medicine, National University of Singapore, Singapore

^4^ Human Potential Translational Research Programme, Yong Loo Lin School of Medicine, National University of Singapore, Singapore, Singapore

^5^ Department of General Practice and Primary Health Care, University of Helsinki, Finland

^6^ Folkhälsan Research Center, Helsinki, Finland

^7^ Departments of Pediatrics, Yong Loo Lin School of Medicine, National University of Singapore, Singapore

^8^ Division of Pediatric Endocrinology, Khoo Teck Puat-National University Children's Medical Institute, National University Hospital, National University Health System, Singapore

^9^ Departments of Pediatrics, and Diagnostic and Interventional Imaging, KK Women's and Children's Hospital, Singapore

^10^ Duke-National University of Singapore Graduate Medical School, Singapore

^11^ Lee Kong Chian School of Medicine, Nanyang Technological University, Singapore

^12^ Saw Swee Hock School of Public Health, National University of Singapore, Singapore

^13^ Singapore Institute for Clinical Sciences (SICS), Agency for Science, Technology and Research (A*STAR), Singapore, Singapore

^14^ Global Centre for Asian Women’s Health, Yong Loo Lin School of Medicine, National University of Singapore

^15^ Bio-Echo Asia Centre for Reproductive Longevity & Equality, Yong Loo Lin School of Medicine, National University of Singapore

^16^ Murdoch Children’s Research Institute, Royal Children’s Hospital, Parkville, VIC, Australia

^17^ Department of Paediatrics, Melbourne University, Parkville, VIC, Australia

^18^ Department of Paediatrics, Monash University, Melbourne, Australia

^19^ Department of Nutrition, Harvard T.H. Chan School of Public Health, Boston, MA, USA

**Additional File 1. Additional cardiometabolic measures**

Among the Nightingale metabolomic data, fatty acids (i.e., total fatty acids, total polyunsaturated fatty acids, total saturated fatty acids and total monounsaturated fatty acids), low-density lipoprotein (LDL), high-density lipoprotein (HDL) cholesterol, triglyceride and inflammation biomarker Glycoprotein acetyls (GlycA) were measured. Venous plasma glucose (mmol/L) was measured using the hexokinase enzymatic method (Abbott Architect c8000 analyzer at KKH and Beckman AU5800 analyzer at NUH). Insulin (mU/L) was measured by a sandwich immunoassay and high-sensitivity C-reactive protein were measured by colorimetry using the Beckman AU5800 analyzer (Beckman Coulter). The homeostasis model assessment of insulin resistance (HOMA1-IR) was calculated as the ratio of fasting insulin to fasting glucose divided by 22.5.

Child peripheral systolic blood pressure (SBP) and diastolic blood pressure (DBP) were measured from the right upper arm (Dinamap CARESCAPE V100, GE Healthcare, Milwaukee, WI) by trained research staff in a quiet room following standardized protocols.^25^ SBP and DBP were measured three times, and the average was used. Carotid intima-media thickness was measured using high-resolution B-mode ultrasound (CX-50 XMatrix, Philips Medical Ultrasound Systems at KKH and Aloka at NUH) at the right common carotid artery 1 cm proximal to the carotid bulb.^25^ The carotid-femoral pulse wave velocity was measured with the child in the supine position using SphygmoCorVx (AtCor Medical, West Ryde, NSW, Australia).^25^ The arterial waveforms of the right carotid and femoral artery were measured, and the pulse wave velocity was calculated as the ratio of the distance traveled by the pulse wave and the time delay between the waveforms.
