# Supplementary material for: Maternal glycemic status during pregnancy and mid-childhood plasma amino acid profiles: findings from a multi-ethnic Asian birth cohort
Source: BMC Med. 2023 Nov 29;21:472. doi: 10.1186/s12916-023-03188-9 (PMC10688057; doi:10.1186/s12916-023-03188-9)
Supplement: Supplementary file 2 — Additional file 2: sFigure 1. Participant flowchart. sFigure 2. Partial spearman correlation analyses of child amino acids and cardiometabolic measures assessed at mid-childhood. sTable 1. Compare participant characteristics with or without amino acids. sTable 2. GUSTO analytic sample cardiometabolic profiles (n = 422). sTable 3. Interaction exploration in liner regression analyses of GDM with child amino acids at mid-childhood. sTable 4. Sensitivity linear regression analyses of fasting glucose with child amino acids at mid-childhood. sTable 5. Sensitivity linear regression analyses of 2-h glucose with child amino acids at mid-childhood. sTable 6. Sensitivity linear regression analyses of GDM with child amino acids at mid-childhood. sTable 7. Sensitivity linear regression analyses of maternal glucose level with child amino acids at mid-childhood with additional adjustment of child birthweight and gestation age. sTable 8. Sensitivity analyses of maternal glucose level with child amino acids at mid-childhood in GDM mothers without medical treatment (n = 413). sTable 9. Associations of maternal glucose level with child amino acids at mid-childhood, model 2 with FDR corrections. [file 12916_2023_3188_MOESM2_ESM.docx]

**Additional file 2**

**Maternal Glycaemic Status during Pregnancy and Mid-Childhood Plasma Amino Acid Profiles: Findings from A Multi-Ethnic Asian Birth Cohort**

Mengjiao Liu, PhD;^1,2^ Shiao-Yng Chan, PhD;^3,4^ Johan G. Eriksson, MD;^3,4,5,6^ Yap Seng Chong, MD;^3^ Yung Seng Lee, PhD;^7,8^ Fabian Yap, MBBS;^9,10,11^ Mary Foong-Fong Chong, PhD;^12^ Mya Thway Tint, PhD;^4,13^ Jiaxi Yang, PhD^3,14,15^ David Burgner, PhD;^16,17,18^ Cuilin Zhang, PhD;^3,4,14,15,19^ Ling-Jun Li, PhD^3,4,14,15*^

**Affiliations:**

^1^ School of Public Health, Nanchang University, Jiangxi, China

^2^ Jiangxi Provincial Key Laboratory of Preventive Medicine, Nanchang University, Jiangxi, China

^3^ Department of Obstetrics & Gynaecology, Yong Loo Lin School of Medicine, National University of Singapore, Singapore

^4^ Human Potential Translational Research Programme, Yong Loo Lin School of Medicine, National University of Singapore, Singapore, Singapore

^5^ Department of General Practice and Primary Health Care, University of Helsinki, Finland

^6^ Folkhälsan Research Center, Helsinki, Finland

^7^ Departments of Pediatrics, Yong Loo Lin School of Medicine, National University of Singapore, Singapore

^8^ Division of Pediatric Endocrinology, Khoo Teck Puat-National University Children's Medical Institute, National University Hospital, National University Health System, Singapore

^9^ Departments of Pediatrics, and Diagnostic and Interventional Imaging, KK Women's and Children's Hospital, Singapore

^10^ Duke-National University of Singapore Graduate Medical School, Singapore

^11^ Lee Kong Chian School of Medicine, Nanyang Technological University, Singapore

^12^ Saw Swee Hock School of Public Health, National University of Singapore, Singapore

^13^ Singapore Institute for Clinical Sciences (SICS), Agency for Science, Technology and Research (A*STAR), Singapore, Singapore

^14^ Global Centre for Asian Women’s Health, Yong Loo Lin School of Medicine, National University of Singapore

^15^ Bio-Echo Asia Centre for Reproductive Longevity & Equality, Yong Loo Lin School of Medicine, National University of Singapore

^16^ Murdoch Children’s Research Institute, Royal Children’s Hospital, Parkville, VIC, Australia

^17^ Department of Paediatrics, Melbourne University, Parkville, VIC, Australia

^18^ Department of Paediatrics, Monash University, Melbourne, Australia

^19^ Department of Nutrition, Harvard T.H. Chan School of Public Health, Boston, MA, USA

# Additional file 2

Fig. S1 Participant flowchart

Table. S1 Compare participant characteristics with or without amino acids

Table. S2 Interaction exploration in liner regression analyses of GDM with child amino acids at mid-childhood.

Table. S3 Sensitivity linear regression analyses of fasting glucose with child amino acids at mid-childhood.

Table. S4 Sensitivity linear regression analyses of 2-hour glucose with child amino acids at mid-childhood.

Table. S5 Sensitivity linear regression analyses of GDM with child amino acids at mid-childhood.

Table. S6 Sensitivity linear regression analyses of maternal glucose level with child amino acids at mid-childhood with additional adjustment of child birthweight and gestation age.

Table. S7 Sensitivity analyses of maternal glucose level with child amino acids at mid-childhood in GDM mothers without medical treatment (n=413).

Table. S8 Associations of maternal glucose level with child amino acids at mid-childhood, model 2 with FDR corrections.

Table. S9 GUSTO analytic sample cardiometabolic profiles (n=422)

Figure. S2 Partial spearman correlation analyses of child amino acids and cardiometabolic measures assessed at mid-childhood

**Fig. S1 Participant flowchart**


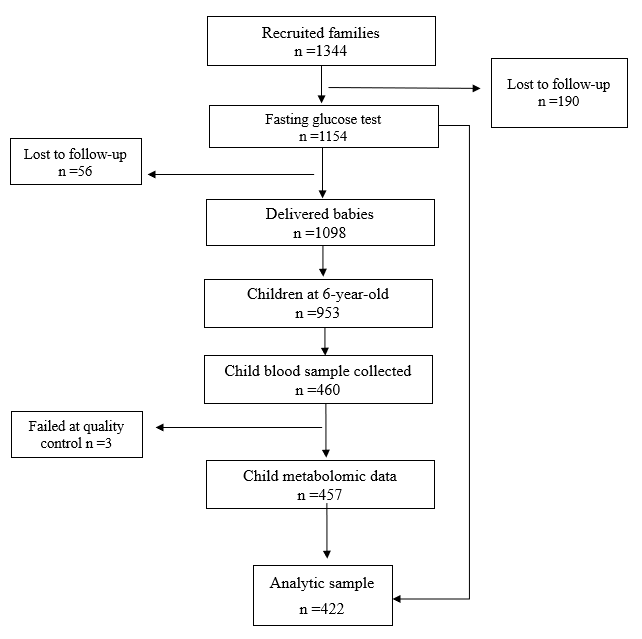


Note: We only consider participants recruited in the Main GUSTO.

**Table. S1. Compare participant characteristics with or without amino acids**

| **Variables** | **With AAs measured**  **(n=422)** | **Without AAs**  **(n=615)** | **p-value** |
| --- | --- | --- | --- |
| ***Mothers*** | **Mean (SD)/ %** | **Mean (SD)/ %** |  |
| **Mother age (whole years)** | **31·0 (5·1)** | **30·0 (5·1)** | **<0·01** |
| Ethnicity |  |  | 0·22 |
| Chinese | 56·4 | 54·5 |  |
| Malay | 27·5 | 25·2 |  |
| Indian | 16·1 | 20·3 |  |
| Highest education |  |  | 0·51 |
| Below university | 64·7 | 66·7 |  |
| University | 35·3 | 33·3 |  |
| **Parity** |  |  | **0·01** |
| **0** | **38·6** | **46·8** |  |
| **≥1** | **61·4** | **53·2** |  |
| Pre-pregnancy BMI (kg/m^2^) | 22·77 (4·25) | 22·6 (4·5) | 0·62 |
| Categorical ppBMI (WHO)* |  |  | 0·77 |
| Normal and underweight | 61·4 | 62·3 |  |
| Overweight or obese | 38·6 | 37·7 |  |
| Total gestational weight gain (kg) | 13·8 (5·2) | 13·7 (5·2) | 0·66 |
| Excessive GWG (according to IOM, %) | 41·6 | 48·2 | 0·99 |
| Glyceamia at 26 weeks’ gestation |  |  |  |
| Fasting glucose, mmol/L | 4·3 (0·5) | 4·4 (0·5) | 0·37 |
| 2-hour fasting glucose, mmol/L | 6·6 (1·5) | 6·5 (1·5) | 0·78 |
| Gestational diabetes (according to 1999 WHO, %) | 19·7 | 18·9 | 0·76 |
| Family history of diabetes (yes, %) | 31·0 | 30·4 | 0·83 |
| Hypertensive disorder (yes, %) | 7·8 | 6·9 | 0·58 |
| **Health eating index** | **53·3** | **51·2 (13·6)** | **<0·05** |
| ***Children*** |  |  |  |
| Child age at year 6 visit | 6·1 (0·1) | 6·06 (·1) | 0·21 |
| Sex (girls, %) | 47·6 | 48·5 | 0·78 |
| Child BMI (kg/m^2^) | 15·5 (2·1) | 15·6 (2·5) | 0·64 |
| Child BMI z-score | -0·04 (1·3) | -0·03 (1·5) | 0·97 |
| Carotid-intima-media thickness (mm) | 0·4 (0·03) | 0·4 (0·03) | 0·83 |
| Pulse wave velocity (m/s) | 4·9 (1·5) | 5·0 (1·7) | 0·36 |
| Augmentation index | 17·3 (10·7) | 19·1 (9·9) | 0·09 |
| Systolic blood pressure (mmHg) | 101·1 (8·1) | 101·3 (8·6) | 0·79 |
| Diastolic blood pressure (mmHg) | 60·1 (5·8) | 59·5 (5·5) | 0·18 |

**Table. S2 Interaction exploration in linear regression analyses of GDM with child amino acids at mid-childhood.**

|  | GDM (Yes)*maternal age (≥35) | | GDM(Yes)*ppBMI cat (OW/OB) | |
| --- | --- | --- | --- | --- |
|  | Β (95%CI) | *p* | Β (95%CI) | *p* |
| Alanine | -2·95 (-34·94, 29·03) | 0·86 | 8·55 (-21·52, 38·63) | 0·58 |
| Glutamine | -22·06 (-55·73, 11·60) | 0·20 | 1·27 (-30·45, 32·99) | 0·94 |
| Glycine | -17·81 (-40·85, 5·23) | 0·13 | -8·46 (-30·19, 13·26) | 0·44 |
| Histidine | 0·60 (-5·22, 6·43) | 0·84 | -3·69 (-9·16, 1·78) | 0·19 |
| Isoleucine | 2·63 (-3·15, 8·42) | 0·37 | -1·76 (-7·20, 3·67) | 0·52 |
| Leucine | 5·52 (-3·22, 14·25) | 0·21 | -1·96 (-10·18, 6·26) | 0·64 |
| Valine | 2·73 (-13·46, 18·93) | 0·74 | -9·50 (-24·69, 5·69) | 0·22 |
| Total BCAA | 10·88 (-17·85, 39·62) | 0·46 | -13·23 (-40·21, 13·76) | 0·34 |
| Phenylalanine | -1·25 (-5·72, 3·23) | 0·58 | -3·03 (-7·21, 1·16) | 0·16 |
| Tyrosine | 1·30 (-4·30, 6·91) | 0·65 | 2·53 (-2·74, 7·80) | 0·35 |
| Aromatic AA | 0·05 (-8·23, 8·34) | 0·99 | -0·50 (-8·31, 7·31) | 0·90 |

Model adjustment: maternal age, child ethnicity, mothers’ highest education, parity, family history of diabetes, child sex, ppBMI, child year 6 BMI z-score and age.

**Table. S3 Sensitivity linear regression analyses of fasting glucose with child amino acids at mid-childhood.**

| Amino acids | Excessive weight gain | | HDP | | Health eating index | | Total fatty acids | | Total PUFAs | | Total SFAs | | Total MUFAs | | Child y5 protein intake | |
| --- | --- | --- | --- | --- | --- | --- | --- | --- | --- | --- | --- | --- | --- | --- | --- | --- |
|  | Β (95%CI) | *p* | Β (95%CI) | *p* | Β (95%CI) | *p* | Β (95%CI) | *p* | Β (95%CI) | *p* | Β (95%CI) | *p* | Β (95%CI) | *p* | Β (95%CI) | *p* |
| Alanine | 1·10 (-12·56, 14·76) | 0·87 | 1·84 (-11·57, 15·25) | 0·79 | 1·77 (-11·75, 15·29) | 0·80 | 0·59 (-12·57, 13·75) | 0·93 | 1·55 (-11·86, 14·95) | 0·82 | 0·07 (-13·01, 13·14) | 0·99 | 0·38 (-12·69, 13·46) | 0·95 | 0·55 (-13·38, 14·48) | 0·94 |
| Glutamine | 8·07 (-6·51, 22·66) | 0·28 | 8·69 (-5·44, 22·83) | 0·23 | 8·36 (-5·89, 22·60) | 0·25 | 8·42 (-5·88, 22·72) | 0·25 | 8·75 (-5·56, 23·07) | 0·23 | 8·48 (-5·84, 22·80) | 0·25 | 8·00 (-6·22, 22·22) | 0·27 | 9·20 (-5·41, 23·82) | 0·22 |
| Glycine | 0·27 (-9·77, 10·31) | 0·96 | 1·11 (-8·56, 10·78) | 0·82 | 0·54 (-9·23, 10·31) | 0·91 | 0·36 (-9·37, 10·09) | 0·94 | 0·72 (-9·05, 10·49) | 0·88 | 0·32 (-9·43, 10·07) | 0·95 | 0·07 (-9·57, 9·72) | 0·99 | 2·84 (-7·25, 12·94) | 0·58 |
| Histidine | 1·32 (-1·24, 3·89) | 0·31 | 1·42 (-1·03, 3·87) | 0·26 | 1·54 (-0·92, 3·99) | 0·22 | 1·03 (-1·40, 3·46) | 0·41 | 1·09 (-1·33, 3·52) | 0·38 | 1·01 (-1·43, 3·45) | 0·42 | 1·06 (-1·38, 3·51) | 0·39 | 1·40 (-1·22, 4·02) | 0·30 |
| Isoleucine | 1·83 (-0·68, 4·35) | 0·15 | 1·77 (-0·65, 4·19) | 0·15 | 1·59 (-0·83, 4·02) | 0·20 | 2·04 (-0·38, 4·45) | 0·10 | 2·07 (-0·33, 4·48) | 0·09 | 2·00 (-0·41, 4·42) | 0·10 | 1·99 (-0·42, 4·41) | 0·11 | 1·63 (-0·94, 4·19) | 0·21 |
| Leucine | 2·00 (-1·83, 5·83) | 0·30 | 1·86 (-1·81, 5·54) | 0·32 | 1·96 (-1·74, 5·66) | 0·30 | 2·30 (-1·39, 5·98) | 0·22 | 2·29 (-1·39, 5·97) | 0·22 | 2·25 (-1·44, 5·94) | 0·23 | 2·30 (-1·39, 5·99) | 0·22 | 1·72 (-2·20, 5·65 | 0·39 |
| Valine | **7·41 (0·39, 14·43)** | **0·04** | **7·74 (0·97, 14·51)** | **0·03** | **8·37 (1·56, 15·17)** | **0·02** | **8·00 (1·20, 14·81)** | **0·02** | **8·04 (1·24, 14·83)** | **0·02** | **7·92 (1·11, 14·72)** | **0·02** | **8·13 (1·32, 14·94)** | **0·02** | 6·37 (-0·95, 13·69) | 0·09 |
| Total BCAA | 11·25 (-1·29, 23·78) | 0·08 | 11·37 (-0·66, 23·41) | 0·06 | **11·92 (-0·19, 24·03)** | **0·05** | **12·34 (0·23, 24·44)** | **0·05** | **12·40 (0·31, 24·50)** | **0·04** | **12·17 (0·07, 24·28)** | **0·05** | **12·42 (0·31, 24·53)** | **0·04** | 9·72 (-3·21, 22·65) | 0·14 |
| Phenylalanine | 0·88 (-1·03, 2·79) | 0·37 | 0·97 (-0·90, 2·84) | 0·31 | 1·11 (-0·77, 3·00) | 0·25 | 1·09 (-0·78, 2·96) | 0·25 | 1·05 (-0·82, 2·92) | 0·27 | 1·10 (-0·77, 2·98) | 0·25 | 1·07 (-0·81, 2·95) | 0·27 | 0·75 (-1·26, 2·76) | 0·46 |
| Tyrosine | 1·10 (-1·34, 3·54) | 0·38 | 1·19 (-1·16, 3·55) | 0·32 | 1·07 (-1·29, 3·44) | 0·37 | 1·38 (-0·99, 3·74) | 0·25 | 1·37 (-0·99, 3·73) | 0·25 | 1·38 (-0·98, 3·75) | 0·25 | 1·36 (-1·00, 3·73) | 0·26 | 0·83 (-1·69, 3·34) | 0·52 |
| Aromatic AA | 2·03 (-1·57, 5·62) | 0·27 | 2·21 (-1·27, 5·69) | 0·21 | 2·23 (-1·27, 5·73) | 0·21 | 2·47 (-1·02, 5·95) | 0·17 | 2·42 (-1·06, 5·91) | 0·17 | 2·49 (-1·00, 5·98) | 0·16 | 2·43 (-1·06, 5·92) | 0·17 | 1·64 (-2·11, 5·38) | 0·39 |

Sensitivity analysis: Model2 + 1. Excessive weight gain; 2. HDP; 3. Maternal health eating index; 4.Total fatty acids; 5. Total polyunsaturated fatty acids (PUFAs); 6. Total saturated fatty acids (SFAs); 7. Total monounsaturated fatty acids (MUFAs).

**Table. S4** **Sensitivity linear regression analyses of 2-hour glucose with child amino acids at mid-childhood.**

| Amino acids | Excessive weight gain | | HDP | | Heath eating index | | Total fatty acids | | Total PUFAs | | Total SFAs | | Total MUFAs | | Child y5 protein intake | |
| --- | --- | --- | --- | --- | --- | --- | --- | --- | --- | --- | --- | --- | --- | --- | --- | --- |
|  | Β (95%CI) | *p* | Β (95%CI) | *p* | Β (95%CI) | *p* | Β (95%CI) | *p* | Β (95%CI) | *p* | Β (95%CI) | *p* | Β (95%CI) | *p* | Β (95%CI) | *p* |
| Alanine | -0·93 (-5·17, 3·31) | 0·67 | -0·80 (-5·01, 3·41) | 0·71 | -1·02 (-5·27, 3·23) | 0·64 | -1·30 (-5·43, 2·82) | 0·54 | -1·09 (-5·30, 3·12) | 0·61 | -1·19 (-5·29, 2·90) | 0·57 | -1·30 (-5·40, 2·79) | 0·53 | -0·08 (-4·56, 4·41) | 0·97 |
| Glutamine | -0·98 (-5·51, 3·56) | 0·67 | -1·63 (-6·07, 2·82) | 0·47 | -1·52 (-6·01, 2·96) | 0·50 | -1·84 (-6·32, 2·65) | 0·42 | -1·70 (-6·20, 2·80) | 0·46 | -1·75 (-6·24, 2·74) | 0·44 | -1·98 (-6·44, 2·48) | 0·38 | -1·88 (-6·59, 2·83) | 0·43 |
| Glycine | -1·34 (-4·46, 1·77) | 0·40 | -1·60 (-4·64, 1·43) | 0·30 | -1·76 (-4·82, 1·31) | 0·26 | -1·90 (-4·95, 1·14) | 0·22 | -1·79 (-4·86, 1·27) | 0·25 | -1·83 (-4·87, 1·22) | 0·24 | -1·99 (-5·01, 1·02) | 0·19 | -0·73 (-3·98, 2·52) | 0·66 |
| Histidine | **1·12 (0·34, 1·91)** | **0·01** | **0·96 (0·20, 1·72)** | **0·01** | **0·87 (0·10, 1·64)** | **0·03** | **0·85 (0·09, 1·61)** | **0·03** | **0·83 (0·08, 1·59)** | **0·03** | **0·87 (0·11, 1·63)** | **0·02** | **0·87 (0·11, 1·63)** | **0·03** | **0·89 (0·05, 1·72)** | **0·04** |
| Isoleucine | **0·87 (0·09, 1·65)** | **0·03** | **0·75 (-0·00, 1·51)** | **0·05** | **0·86 (0·10, 1·62)** | **0·03** | **0·84 (0·08, 1·60)** | **0·03** | **0·88 (0·12, 1·63)** | **0·02** | **0·83 (0·07, 1·58)** | **0·03** | **0·82 (0·07, 1·58)** | **0·03** | 0·77 (-0·06, 1·59) | 0·07 |
| Leucine | **1·61 (0·44, 2·79)** | **0·01** | **1·57 (0·42, 2·71)** | **0·01** | **1·68 (0·53, 2·83)** | **0·00** | **1·63 (0·48, 2·78** | **0·01** | **1·65 (0·51, 2·80)** | **0·00** | **1·61 (0·46, 2·75)** | **0·01** | **1·63 (0·48, 2·77)** | **0·01** | **1·65 (0·40, 2·90)** | **0·01** |
| Valine | **3·08 (0·91, 5·25)** | **0·01** | **2·71 (0·59, 4·83)** | **0·01** | **2·73 (0·59, 4·87)** | **0·01** | **2·72 (0·59, 4·85)** | **0·01** | **2·70 (0·57, 4·83)** | **0·01** | **2·72 (0·59, 4·84)** | **0·01** | **2·78 (0·65, 4·91)** | **0·01** | **2·71 (0·36, 5·06)** | **0·02** |
| Total BCAA | **5·56 (1·70, 9·43)** | **0·00** | **5·03 (1·26, 8·79)** | **0·01** | **5·27 (1·48, 9·06)** | **0·01** | **5·19 (1·41, 8·97)** | **0·01** | **5·23 (1·45, 9·01)** | **0·01** | **5·15 (1·38, 8·93)** | **0·01** | **5·23 (1·45, 9·01)** | **0·01** | **5·12 (0·99, 9·26)** | **0·02** |
| Phenylalanine | **0·67 (0·08, 1·26)** | **0·03** | 0·52 (-0·06, 1·11) | 0·08 | 0·54 (-0·05, 1·13) | 0·07 | 0·55 (-0·04,1·13) | 0·07 | 0·56 (-0·03, 1·14) | 0·06 | 0·53 (-0·05, 1·12) | 0·07 | 0·53 (-0·06, 1·12) | 0·08 | 0·57 (-0·08, 1·21) | 0·08 |
| Tyrosine | 0·34 (-0·42, 1·09) | 0·38 | 0·34 (-0·40, 1·08) | 0·36 | 0·40 (-0·34, 1·15) | 0·29 | 0·39 (-0·35, 1·13) | 0·30 | 0·40 (-0·35, 1·14) | 0·29 | 0·39 (-0·35, 1·13) | 0·30 | 0·38 (-0·36, 1·12) | 0·31 | 0·29 (-0·51, 1·10) | 0·47 |
| Aromatic AA | 1·01 (-0·09, 2·12) | 0·07 | 0·87 (-0·21, 1·96) | 0·11 | 0·95 (-0·14, 2·05) | 0·09 | 0·94 (-0·16, 2·03) | 0·09 | 0·95 (-0·14, 2·04) | 0·09 | 0·92 (-0·17, 2·01) | 0·10 | 0·91 (-0·18, 2·01) | 0·10 | 0·88 (-0·32, 2·08) | 0·15 |

Sensitivity analysis: Model2 + 1. Excessive weight gain; 2. HDP; 3. Maternal health eating index; 4.Total fatty acids; 5. Total polyunsaturated fatty acids (PUFAs); 6. Total saturated fatty acids (SFAs); 7. Total monounsaturated fatty acids (MUFAs).

**Table. S5 Sensitivity liner regression analyses of GDM with child amino acids at mid-childhood.**

| Amino acids | Excessive weight gain | | HDP | | Health eating index | | Total fatty acids | | Total PUFAs | | Total SFAs | | Total MUFAs | | Child y5 protein intake | |
| --- | --- | --- | --- | --- | --- | --- | --- | --- | --- | --- | --- | --- | --- | --- | --- | --- |
|  | Β (95%CI) | *p* | Β (95%CI) | *p* | Β (95%CI) | *p* | Β (95%CI) | *p* | Β (95%CI) | *p* | Β (95%CI) | *p* | Β (95%CI) | *p* | Β (95%CI) | *p* |
| Alanine | -7·85 (-23·24, 7·55) | 0·32 | -7·43 (-23·00, 8·14) | 0·35 | -8·84 (-24·48, 6·80) | 0·27 | -10·03 (-25·23, 5·17) | 0·20 | -8·52 (-24·00, 6·96) | 0·28 | -10·00 (-25·08, 5·09) | 0·19 | -10·94 (-26·05, 4·16) | 0·16 | -9·30 (-25·60, 7·00) | 0·26 |
| Glutamine | -3·97 (-20·46, 12·51) | 0·64 | -6·41 (-22·86, 10·04) | 0·44 | -6·08 (-22·60, 10·44) | 0·47 | -7·00 (-23·56, 9·56) | 0·41 | -6·37 (-22·94, 10·20) | 0·45 | -6·73 (-23·30, 9·84) | 0·42 | -8·04 (-24·52, 8·44) | 0·34 | -6·57 (-23·73, 10·59) | 0·45 |
| Glycine | -7·79 (-19·09, 3·52) | 0·18 | -7·75 (-18·96, 3·46) | 0·17 | -8·89 (-20·17, 2·39) | 0·12 | -9·04 (-20·26, 2·18) | 0·11 | -8·42 (-19·69, 2·85) | 0·14 | -8·84 (-20·08, 2·39) | 0·12 | -9·83 (-20·96, 1·30) | 0·08 | -2·42 (-14·26, 9·42) | 0·69 |
| Histidine | 2·05 (-0·83, 4·93) | 0·16 | 2·08 (-0·74, 4·91) | 0·15 | 1·62 (-1·22, 4·45) | 0·26 | 1·49 (-1·33, 4·30) | 0·30 | 1·55 (-1·26, 4·35) | 0·28 | 1·54 (-1·28, 4·36) | 0·28 | 1·51 (-1·32, 4·34 | 0·30 | 2·19 (-0·87, 5·24) | 0·16 |
| Isoleucine | 1·97 (-0·87, 4·81) | 0·17 | 1·40 (-1·41, 4·22) | 0·33 | 1·83 (-0·98, 4·64) | 0·20 | 1·69 (-1·11, 4·49) | 0·24 | 1·78 (-1·01, 4·56) | 0·21 | 1·64 (-1·16, 4·43) | 0·25 | 1·59 (-1·21, 4·39) | 0·26 | 1·98 (-1·02, 4·98) | 0·20 |
| Leucine | **4·83 (0·53, 9·13)** | **0·03** | **4·37 (0·11, 8·62)** | **0·04** | **4·93 (0·66, 9·19)** | **0·02** | **4·60 (0·35, 8·85)** | **0·03** | **4·61 (0·37, 8·85)** | **0·03** | **4·51 (0·26, 8·76)** | **0·04** | **4·63 (0·38, 8·89)** | **0·03** | **5·43 (0·86, 9·99)** | **0·02** |
| Valine | **9·45 (1·54, 17·36)** | **0·02** | **8·01 (0·13, 15·88)** | **0·05** | **8·16 (0·26, 16·06)** | **0·04** | **7·78 (0·11, 15·67)** | **0·05** | **7·80 (0·08, 15·67)** | **0·05** | **7·72 (0·15, 15·60)** | **0·05** | **8·02 (0·12, 15·93)** | **0·05** | **9·33 (0·77, 17·88)** | **0·03** |
| Total BCAA | **16·25 (2·14, 30·36)** | **0·02** | **13·78 (-0·20, 27·76)** | **0·05** | **14·92 (0·90, 28·94)** | **0·04** | **14·07 (0·06, 28·08)** | **0·05** | **14·18 (0·19, 28·18)** | **0·05** | **13·87 (-0·13, 27·86)** | **0·05** | **14·25 (0·22, 28·27)** | **0·05** | **16·73 (1·64, 31·83)** | **0·03** |
| Phenylalanine | 1·45 (-0·71, 3·60) | 0·19 | 0·82 (-1·35, 2·99) | 0·46 | 0·95 (-1·23, 3·14) | 0·39 | 0·93 (-1·24, 3·10) | 0·40 | 0·89 (-1·27, 3·06) | 0·42 | 0·90 (-1·27, 3·07) | 0·42 | 0·91 (-1·27, 3·08) | 0·41 | 1·25 (-1·11, 3·61) | 0·30 |
| Tyrosine | -0·28 (-3·03, 2·46) | 0·84 | -0·35 (-3·08, 2·38) | 0·80 | -0·14 (-2·87, 2·60) | 0·92 | -0·11 (-2·85, 2·63) | 0·94 | -0·11 (-2·85, 2·63) | 0·94 | -0·11 (-2·85, 2·63) | 0·94 | -0·13 (-2·88, 2·61) | 0·92 | -0·46 (-3·39, 2·48) | 0·76 |
| Aromatic AA | 1·17 (-2·88, 5·21) | 0·57 | 0·47 (-3·57, 4·50) | 0·82 | 0·81 (-3·24, 4·86) | 0·69 | 0·82 (-3·23, 4·86) | 0·69 | 0·78 (-3·25, 4·82) | 0·70 | 0·79 (-3·26, 4·83) | 0·70 | 0·77 (-3·28, 4·83) | 0·71 | 0·79 (-3·58, 5·17) | 0·72 |

Sensitivity analysis: Model2 + 1. Excessive weight gain; 2. HDP; 3. Maternal health eating index; 4.Total fatty acids; 5. Total polyunsaturated fatty acids (PUFAs); 6. Total saturated fatty acids (SFAs); 7. Total monounsaturated fatty acids (MUFAs).

**Table. S6 Sensitivity linear regression analyses of maternal glucose level with child amino acids at mid-childhood with additional adjustment of child birthweight and gestation age.**

| **Child amino acids measured at mid-childhood** | **Fasting glucose** | | **Two-hour glucose** | | **Gestational diabetes** | |
| --- | --- | --- | --- | --- | --- | --- |
|  | **Effect size (95%CI)** | **p** | **Effect size (95%CI)** | **p** | **Effect size (95%CI)** | **p** |
| Alanine | 0.03 (-0.07, 0.13) | 0.56 | -0.01 (-0.11, 0.09) | 0.81 | -0.12 (-0.37, 0.13) | 0.35 |
| Glutamine | 0.08 (-0.02, 0.18) | 0.12 | -0.03 (-0.13, 0.07) | 0.56 | -0.08 (-0.33, 0.17) | 0.53 |
| Glycine | -0.00 (-0.11, 0.10) | 0.93 | -0.06 (-0.17, 0.04) | 0.24 | -0.20 (-0.46, 0.06) | 0.13 |
| Histidine | 0.08 (-0.03, 0.18) | 0.17 | **0.14 (0.03, 0.24)** | **0.01** | 0.20 (-0.06, 0.46) | 0.14 |
| Isoleucine | 0.10 (-0.01, 0.20) | 0.07 | **0.11 (0.01, 0.22)** | **0.03** | 0.15 (-0.10, 0.41) | 0.24 |
| Leucine | 0.09 (-0.01, 0.20) | 0.08 | **0.16 (0.06, 0.26)** | **0.00** | **0.31 (0.06, 0.56)** | **0.02** |
| Valine | **0.16 (0.06, 0.26)** | **0.00** | **0.15 (0.05, 0.25)** | **0.00** | **0.30 (0.05, 0.55)** | **0.02** |
| Total BCAAs | **0.14 (0.03, 0.24)** | **0.01** | **0.15 (0.05, 0.26)** | **0.00** | **0.30 (0.04, 0.55)** | **0.02** |
| Phenylalanine | 0.05 (-0.05, 0.15) | 0.29 | 0.09 (-0.01, 0.19) | 0.08 | 0.11 (-0.13, 0.36) | 0.36 |
| Tyrosine | 0.06 (-0.04, 0.16) | 0.22 | 0.05 (-0.05, 0.15) | 0.31 | -0.02 (-0.26, 0.23) | 0.88 |
| Aromatic AA | 0.07 (-0.03, 0.17) | 0.16 | 0.08 (-0.02, 0.18) | 0.10 | 0.05 (-0.19, 0.29) | 0.70 |

Adjusted covariates: maternal age, child ethnicity, mothers’ highest education, parity, family history of diabetes, child sex, pre-pregnancy BMI child year 6 body mass index z-score, age at measurement, birthweight and gestational age at birth.

**Table. S7 Sensitivity analyses of maternal glucose level with child amino acids at mid-childhood in GDM mothers without medical treatment (n=413).**

| ***Child amino acids*** | **GDM vs. non-GDM (reference)** | |
| --- | --- | --- |
|  | β (95%CI) | *p* |
| Alanine | -10·65 (-26·72, 5·42) | 0·19 |
| Glutamine | -6·22 (-23·09, 10·66) | 0·47 |
| Glycine | -7·28 (-18·94, 4·38) | 0·22 |
| Histidine | 2·46 (-0·46, 5·38) | 0·10 |
| Isoleucine | 1·28 (-1·65, 4·20) | 0·39 |
| Leucine | 4·63 (0·22, 9·04) | 0·04 |
| Valine | 7·26 (-0·92, 15·44) | 0·08 |
| Total BCAAs | 13·17 (-1·37, 27·70) | 0·08 |
| Phenylalanine | 0·92 (-1·34, 3·18) | 0·42 |
| Tyrosine | -0·16 (-3·00, 2·68) | 0·91 |
| Aromatic AA | 0.76 (-3.44, 4.96) | 0.72 |

Model adjusted for: maternal age, child ethnicity, mothers’ highest education, parity, family history of diabetes, child sex and pre-pregnancy BMI

**Table. S8 Associations of maternal glucose level with child amino acids at mid-childhood, model 2 with FDR corrections.**

| **Child amino acids measured at mid-childhood** | **Fasting glucose** | | | **Two-hour glucose** | | | **Gestational diabetes** | | |
| --- | --- | --- | --- | --- | --- | --- | --- | --- | --- |
|  | **Effect size (95%CI)** | **p** | **FDR correction** | **Effect size (95%CI)** | **p** | **FDR correction** | **Effect size (95%CI)** | **p** | **FDR correction** |
| Alanine | 0.01 (-0.09, 0.11) | 0.87 | 0.93 | -0.02 (-0.12, 0.08) | 0.67 | 0.67 | -0.14 (-0.39, 0.11) | 0.28 | 0.47 |
| Glutamine | 0.06 (-0.04, 0.16) | 0.23 | 0.38 | -0.04 (-0.14, 0.06) | 0.47 | 0.52 | -0.10 (-0.35, 0.15) | 0.44 | 0.54 |
| Glycine | 0.00 (-0.10, 0.11) | 0.93 | 0.93 | -0.06 (-0.16, 0.05) | 0.27 | 0.37 | -0.20 (-0.46, 0.06) | 0.13 | 0.36 |
| Histidine | 0.06 (-0.05, 0.16) | 0.30 | 0.38 | **0.13 (0.02, 0.23)** | **0.02** | 0.06 | 0.18 (-0.08, 0.44) | 0.18 | 0.40 |
| Isoleucine | 0.08 (-0.03, 0.18) | 0.14 | 0.38 | 0.10 (0.00, 0.21) | 0.05 | 0.11 | 0.14 (-0.12, 0.39) | 0.30 | 0.47 |
| Leucine | 0.06 (-0.04, 0.16) | 0.27 | 0.38 | **0.14 (0.04, 0.24)** | **0.01** | **0.04** | **0.28 (0.02, 0.53)** | **0.03** | 0.15 |
| Valine | **0.12 (0.02, 0.22)** | **0.02** | 0.22 | **0.13 (0.03, 0.23)** | **0.01** | **0.04** | **0.27 (0.01, 0.52)** | **0.04** | 0.15 |
| Total BCAAs | 0.10 (-0.00, 0.20) | 0.05 | 0.28 | **0.14 (0.04, 0.24)** | **0.01** | **0.04** | **0.26 (0.01, 0.52)** | **0.04** | 0.15 |
| Phenylalanine | 0.06 (-0.04, 0.15) | 0.26 | 0.38 | 0.09 (-0.01, 0.19) | 0.07 | 0.13 | 0.11 (-0.13, 0.36) | 0.37 | 0.51 |
| Tyrosine | 0.05 (-0.05, 0.15) | 0.31 | 0.38 | 0.05 (-0.05, 0.14) | 0.36 | 0.44 | -0.03 (-0.27, 0.21) | 0.82 | 0.82 |
| Aromatic AA | 0.06 (-0.03, 0.16) | 0.19 | 0.38 | 0.08 (-0.02, 0.18) | 0.11 | 0.17 | 0.04 (-0.20, 0.28) | 0.74 | 0.81 |

Abbreviations: FDR, false-discovery rate; AA, amino acids.

Adjusted covariates: maternal age, child ethnicity, mothers’ highest education, parity, family history of diabetes, child sex, pre-pregnancy BMI child year 6 body mass index z-score, and age at measurement.

**Table. S9 GUSTO analytic sample cardiometabolic profiles (n=422)**

| **Variables** | **All children** | **Born to mother with GDM** | **Born to mother without GDM** | **T-test p-value** |
| --- | --- | --- | --- | --- |
| Total fatty acids (mmol/L) | 114·0 (16·7) | 10·7 (1·3) | 10·6 (1·2) | 0·90 |
| Polyunsaturated fatty acids (mmol/L) | 10·6 (1·2) | 4·6 (0·5) | 4·5 (0·5) | 0·61 |
| Monounsaturated fatty acids (mmol/L) | 4·5 (0·5) | 2·5 (0·5) | 2·5 (0·4) | 0·93 |
| Saturated fatty acids (mmol/L) | 2·5 (0·4) | 3·6 (0·5) | 3·6 (0·4) | 0·91 |
| HOMA-IR | 0·9 (0·6) | 0·8 (0·5) | 0·9 (0·7) | 0·56 |
| C-reactive protein (mg/L) | 1·2 (2·8) | 0·9 (1·7) | 1·2 (2·7) | 0·48 |
| Total cholesterol (mmol/L) | 4·3 (0·7) | 4·1 (0·7) | 4·3 (0·7) | 0·05 |
| High-density lipoprotein cholesterol (mmol/L) | 1·4 (0·3) | 1·4 (0·3) | 1·4 (0·3) | 0·36 |
| **Low-density lipoprotein cholesterol (mmol/L)** | **2·5 (0·6)** | **2·4 (0·6)** | **2·6 (0·6)** | **0·04** |
| Triglyceride (mmol/L) | 0·7 (0·3) | 0·8 (0·3) | 0·7 (0·3) | 0·42 |
| Carotid-intima-media thickness (mm) | 0·4 (0·03) | 0·4 (0·03) | 0·4 (0·03) | 0·13 |
| Pulse wave velocity (m/s) | 4·9 (1·5) | 4·9 (1·3) | 4·9 (1·5) | 0·82 |
| Augmentation index | 17·3 (10·7) | 19·8 (8·7) | 16·8 (11·0) | 0·06 |
| Systolic blood pressure (mmHg) | 101·1 (8·1) | 101·8 (7·9) | 100·8 (8·1) | 0·38 |
| Diastolic blood pressure (mmHg) | 60·1 (5·8) | 60·3 (6·0) | 60·1 (5·8) | 0·78 |

**Fig. S2 Partial spearman correlation analyses of child amino acids and cardiometabolic measures assessed at mid-childhood**


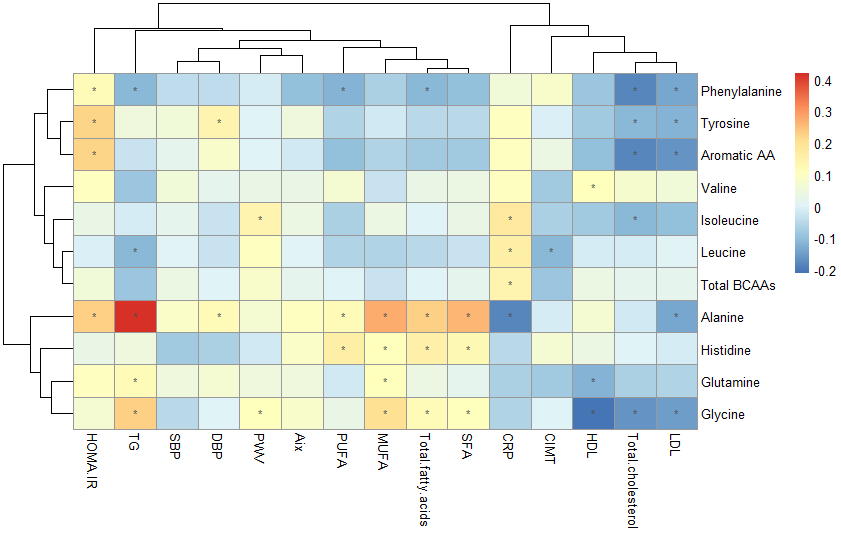


Red color represents positive correlation and blue represents negative correlations, with darker gradient of higher correlations. Stars indicate statistical significance of p<0.05. Model was adjusted for maternal age, ppBMI, GDM status, GWG, child sex, age and BMI z-score at mid-childhood.

Abbreviations: BCAAs, total branched-chain amino acids; Aromatic AA, aromatic amino acids; BMI, body mass index; PUFA, Polyunsaturated fatty acids; MUFA, Monounsaturated fatty acids; SFA, Saturated fatty acids; HOMA-IR, homeostasis model assessment for insulin resistance; CRP, C-reactive protein; HDL, high-density lipoprotein cholesterol; LDL, low-density lipoprotein cholesterol; TG, triglyceride; CIMT, carotid-intima-media thickness; PWV, pulse wave velocity; AIx, Augmentation index; SBP, systolic blood pressure; DBP, diastolic blood pressure.
